# KIAA1429 promotes gastric cancer progression by destabilizing *RASD1* mRNA in an m^6^A-YTHDF2-dependent manner

**DOI:** 10.1186/s12967-024-05375-5

**Published:** 2024-06-20

**Authors:** Mengting Ren, Hanghai Pan, Xinxin Zhou, Mosang Yu, Feng Ji

**Affiliations:** 1 Cancer Center, Department of Gastroenterology, Zhejiang Provincial People’s Hospital (Affiliated People’s Hospital), Hangzhou Medical College, Hangzhou, Zhejiang China; 2 Department of Gastroenterology, The First Affiliated Hospital, Zhejiang University School of Medicine, 79 Qingchun Road, Hangzhou, 310003 Zhejiang China

**Keywords:** KIAA1429, Gastric cancer, m^6^A, RASD1, mRNA stability, YTHDF2

## Abstract

**Background:**

KIAA1429, a regulatory subunit of the N^6^-methyladenosine (m^6^A) methyltransferase complex, has been implicated in the progression of various cancers. However, the role of KIAA1429 in gastric cancer (GC) and its underlying mechanisms remain elusive. This study aimed to investigate the role of KIAA1429 in GC and to elucidate the underlying mechanisms.

**Methods:**

The expression patterns and clinical relevance of KIAA1429 in GC were assessed using quantitative real-time PCR (qRT-PCR), Western blotting, immunohistochemistry (IHC), and bioinformatic analysis. In vitro and in vivo loss- and gain-of-function assays, m^6^A dot blot assays, methylated RNA immunoprecipitation sequencing (MeRIP-seq), RNA-seq, MeRIP-qPCR, dual luciferase reporter assays, RNA stability assays, RNA immunoprecipitation (RIP) assays, and RNA pull-down assays were performed to investigate the biological functions and underlying molecular mechanisms of KIAA1429 in GC.

**Results:**

Both the mRNA and protein expression of KIAA1429 were greater in GC tissues than in normal gastric tissues. High KIAA1429 expression correlated positively with poor prognosis in GC patients. KIAA1429 not only promoted GC cell proliferation, colony formation, G2/M cell cycle transition, migration, and invasion in vitro but also enhanced GC tumor growth and metastasis in vivo. Mechanistically, KIAA1429 increased the m^6^A level of *RASD1* mRNA and enhanced its stability in an m^6^A-YTHDF2-dependent manner, thereby upregulating its expression. RASD1 knockdown partially rescued the KIAA1429 knockdown-induced impairment of pro‑oncogenic ability in GC cells. The expression levels of KIAA1429 and RASD1 were negatively correlated in GC tissues.

**Conclusions:**

KIAA1429 plays a pro‑oncogenic role in GC by downregulating RASD1 expression through destabilizing *RASD1* mRNA in an m^6^A-YTHDF2-dependent manner. KIAA1429 may serve as a prognostic biomarker and therapeutic target for GC.

**Supplementary Information:**

The online version contains supplementary material available at 10.1186/s12967-024-05375-5.

## Introduction

Gastric cancer (GC) ranks as the fifth most common cancer and the third leading cause of cancer mortality globally, with the highest incidence recorded in East Asia [1]. Despite considerable efforts over decades to develop novel treatment modalities, the clinical outcomes for GC patients remain unsatisfactory [2, 3]. Consequently, there is an urgent need to unravel the molecular mechanisms underlying GC development and progression and to identify therapeutic targets for personalized, targeted therapies.

N^6^-methyladenosine (m^6^A) is the most widely distributed RNA modification in eukaryotes, accounting for 0.2–0.6% of all adenosines in mammalian mRNA [4–6]. The m^6^A modification represents a dynamic and reversible biological process orchestrated by methyltransferase (writer), demethylase (eraser), and effector (reader) proteins [4]. Dysregulated m^6^A modification and its associated regulatory proteins have been implicated in important roles in GC tumorigenicity, proliferation, drug resistance, and metastasis, primarily by regulating RNA metabolism, including splicing, degradation, and translation [7–10]. Moreover, several m^6^A regulatory proteins have emerged as potential biomarkers for predicting the prognosis of GC patients. Notably, elevated expression of METTL3, WTAP, and FTO in GC is associated with poor overall survival (OS) [11–13], whereas high METTL14 expression is correlated with prolonged survival [14].

Vir-like m^6^A methyltransferase associated (VIRMA, also known as KIAA1429) is the largest known protein within the methyltransferase complex and is capable of recruiting catalytic core components (METTL3/METTL14/WTAP) to guide regioselective m^6^A modification [15]. KIAA1429-knockdown cells exhibit significantly lower m^6^A levels than METTL3-, METTL14- or WTAP-knockdown cells, highlighting the critical role of KIAA1429 in m^6^A modification [16]. Recent studies have implicated KIAA1429 in the development and progression of various cancers through both m^6^A-depenent [17–20] and m^6^A-independent mechanisms [21, 22]. Miao et al. reported that KIAA1429 could promote GC proliferation by stabilizing *c-Jun* mRNA in an m^6^A‐independent manner [23]. Nevertheless, the precise biological role of KIAA1429 in GC and its molecular mechanisms remain incompletely elucidated.

In the present study, we aimed to investigate the role of KIAA1429 in GC and elucidate the underlying mechanisms involved. We observed that KIAA1429 is upregulated in GC and is associated with poor survival. In vitro and in vivo experiments demonstrated that KIAA1429 promotes GC proliferation and metastasis. Mechanistically, KIAA1429 stabilized the downstream tumor suppressor *RASD1* mRNA in an m^6^A-YTHDF2-dependent manner, thereby upregulating RASD1 expression. Our data suggest that KIAA1429 is a potential prognostic biomarker and a promising target for GC treatment.

## Materials and methods

### Patients and clinical samples

A total of 110 GC specimens, along with paired adjacent noncancer gastric tissues, were obtained from patients undergoing radical gastrectomy at the First Affiliated Hospital, Zhejiang University School of Medicine. Additionally, a human GC tissue microarray consisting of 86 GC specimens and 79 normal gastric tissues was purchased from Outdo Biotech (HStmA180Su16, Shanghai, China). This study adhered to the principles outlined in the Declaration of Helsinki and received approval from the Ethics Committee of the First Affiliated Hospital, Zhejiang University School of Medicine. Written informed consent was obtained from all patients before collection.

### Cell lines and cell culture

Human GC cell lines (AGS, MGC-803, HGC-27, KATO III, MKN-45, and NCI-N87) and an immortalized normal gastric epithelial cell line, GES-1, were obtained from the Cell Bank of the Chinese Academy of Sciences (Shanghai, China). MGC-803, HGC-27, MKN-45, and NCI-N87 cells were cultured in RPMI-1640 medium (Sigma‒Aldrich, Darmstadt, Germany) supplemented with 10% fetal bovine serum (FBS; Sigma‒Aldrich) and 1% penicillin‒streptomycin. AGS and KATO III cells were cultured in Ham’s F-12 K (Kaighn’s) medium (Gibco, Carlsbad, CA, USA) and IMDM (Gibco), respectively, containing 10% FBS and 1% penicillin‒streptomycin. All cells were maintained at 37 °C in a humidified 5% CO_2_ atmosphere.

### In vivo animal studies

Female BALB/c nude mice (4 weeks old) were purchased from Ziyuan Biotechnology (Hangzhou, China). The mice were housed under specific pathogen-free (SPF) conditions and acclimatized for 1 week before the experiments. All animal experiments were approved by the Ethics Committee of The First Affiliated Hospital, Zhejiang University School of Medicine. For KIAA1429 knockdown experiments in tumor-bearing nude mice, HGC-27 cells (1 × 10^7^) were suspended in 200 µL of serum-free RPMI-1640 medium and subcutaneously injected into each mouse. For the rescue experiments, HGC-27 cells (1 × 10^7^) were suspended in 100 µL of serum-free RPMI-1640 medium, gently mixed with 100 µL of Matrigel (Corning, NY, USA), and then injected subcutaneously. Tumor size was measured every 3 days using a caliper, and tumor volume was calculated using the following formula: Volume = length×width^2^/2. For lung metastasis assays, MKN-45 cells (1 × 10^6^) in 100 µL of PBS were intravenously injected into the mice. Approximately 1 month after cell injection, the mice were sacrificed, and the tumors and lung tissues were harvested for subsequent experiments.

### Generation of stable knockdown and overexpression cell lines

Lentiviruses for KIAA1429 knockdown and overexpression were obtained from GeneChem (Shanghai, China). Lentiviruses combined with HitransG A or HitransG G reagent (GeneChem) were added to GC cells at a multiplicity of infection (MOI) of 20, and the cells were incubated for 72 h. The infected cells were then cultured in medium supplemented with 3 µg/mL puromycin (MCE, Shanghai, China) for 5 days for selection.

### siRNA and plasmid transfection

Specific siRNAs and overexpressing plasmids were designed and synthesized by GenePharma (Shanghai, China). GC cells were seeded into 6-well or 12-well plates and cultured until they reached 50% confluence. siRNA and plasmid transfections were then carried out using Lipofectamine 3000 (Invitrogen, Waltham, MA, USA) following the manufacturer’s protocol. The cells were collected at the indicated time points and analyzed.

### Quantification of m^6^A

The m^6^A levels of total RNA in GC tissues and cells were colorimetrically measured using the EpiQuik m^6^A RNA Methylation Quantification Kit (Epigentek, Farmingdale, NY, USA) according to the manufacturer’s instructions. Briefly, 200 ng of total RNA was added to each assay well, and diluted capture antibody and detection antibody were successively added. The absorbance of each well was measured at 450 nm to calculate the m^6^A levels.

### m^6^A dot blot

mRNA was enriched using the Dynabeads mRNA Purification Kit (Invitrogen) and diluted to final concentrations of 400 ng/µL and 200 ng/µL. After denaturation by heating at 95 °C for 3 min, 2 µL of each RNA sample was spotted onto a nylon membrane (Logan, UT, USA). The RNA was UV crosslinked to the nylon membrane. The membranes were then blocked with 5% skim milk and incubated with an anti-m^6^A antibody (1∶2,000, Synaptic Systems, Gottingen, Germany) overnight at 4 °C. Subsequently, the membranes were washed and incubated with a secondary antibody (goat anti-rabbit HRP-conjugated antibody, Solarbio), and detection was performed using enhanced chemiluminescence (ECL) reagent (FUDE, Hangzhou, China). Methylene blue staining was used as the loading control.

### Methylated RNA immunoprecipitation sequencing (MeRIP-seq) and MeRIP-qPCR

Methylated RNA immunoprecipitation (MeRIP) was performed using the Magna MeRIP m^6^A Kit (Millipore) following the manufacturer’s instructions. Total RNA was extracted, and mRNA was enriched using the Dynabeads mRNA Purification Kit (Invitrogen). Subsequently, the RNA was chemically fragmented to 200–300 nucleotides in length and purified with an RNase MiniElute Kit (Qiagen, Germantown, MD, USA). These mRNA fragments were then immunoprecipitated with 5 µg of anti-m^6^A antibody, and the RNA was eluted from the beads. After further purification using the RNase MiniElute Kit (Qiagen), the RNA was subjected to MeRIP-seq and qRT‒PCR. MeRIP‐seq and mRNA sequencing (mRNA‐seq) were performed at LC BioTechnology Co., Ltd. (Hangzhou, China) using an Illumina NovaSeq 6000 sequencer (Illumina, San Diego, CA, USA).

### Dual-luciferase reporter assay

To identify m^6^A sites on *RASD1* mRNA, we constructed wild-type (WT) and mutant (MUT) plasmids containing the *RASD1* 3′ UTR. GC cells were cotransfected with KIAA1429 knockdown or control plasmids and luciferase reporter plasmids using Lipofectamine 3000 (Invitrogen) following the manufacturer’s instructions. After a 24 h incubation, luciferase activity was determined and normalized to Renilla luciferase activity using the Dual Glo Luciferase Assay System (Promega, Madison, WI, USA).

### RNA stability assay

To assess the half-life of *RASD1* mRNA in GC cells, actinomycin D (Act D, 10 µg/mL, MCE) was added to cells and incubated for 0, 1, 2, and 3 h for AGS cells and 0, 2, 4, and 6 h for HGC-27 cells. Subsequently, total RNA was harvested at the indicated times for analysis of the remaining *RASD1* mRNA using qRT‒PCR.

### RNA immunoprecipitation (RIP) assay

The RNA immunoprecipitation (RIP) assay was conducted using the Magna RIP RNA-binding protein immunoprecipitation kit (Millipore) following the manufacturer’s instructions. Briefly, GC cells (2 × 10^7^) were lysed with RIP lysis buffer containing protease and RNase inhibitors. Subsequently, the samples were subjected to immunoprecipitation with the anti-KIAA1429 antibody (Cell Signaling Technology) or nonimmunized rabbit IgG. The RNA‒protein complexes were incubated with proteinase K, and the isolated RNA was validated by qRT‒PCR to assess enrichment.

### RNA pull-down assay

The RNA pull-down assay was performed using the Magnetic RNA–Protein Pull-Down Kit (Thermo Fisher Scientific, Waltham, MA, USA) according to the manufacturer’s instructions. Briefly, biotin-labeled RASD1 RNA was incubated with streptavidin magnetic beads, and then the RNA-bound beads were incubated with GC cell lysates. Finally, the beads were magnetically separated, washed, and boiled in sodium dodecyl sulfate (SDS) buffer to elute proteins for Western blot analysis.

### Statistical analysis

The data are presented as the mean ± standard deviation (SD). Statistical analyses were performed using GraphPad Prism version 9.0 (GraphPad Software, La Jolla, CA, USA) and SPSS version 26.0 (SPSS, Chicago, IL, USA). Variables were compared using Student’s *t* test, Wilcoxon rank-sum test, one-way analysis of variance, Dunnett *t* test, or chi-squared test as appropriate. Pearson correlation was used to assess the correlation between mRNA expression. Survival analyses were performed using Kaplan‒Meier curves and log-rank tests. A two-tailed *P* value < 0.05 was considered to indicate statistical significance.

## Results

### KIAA1429 upregulation in GC and its correlation with poor prognosis

**Fig. 1:**
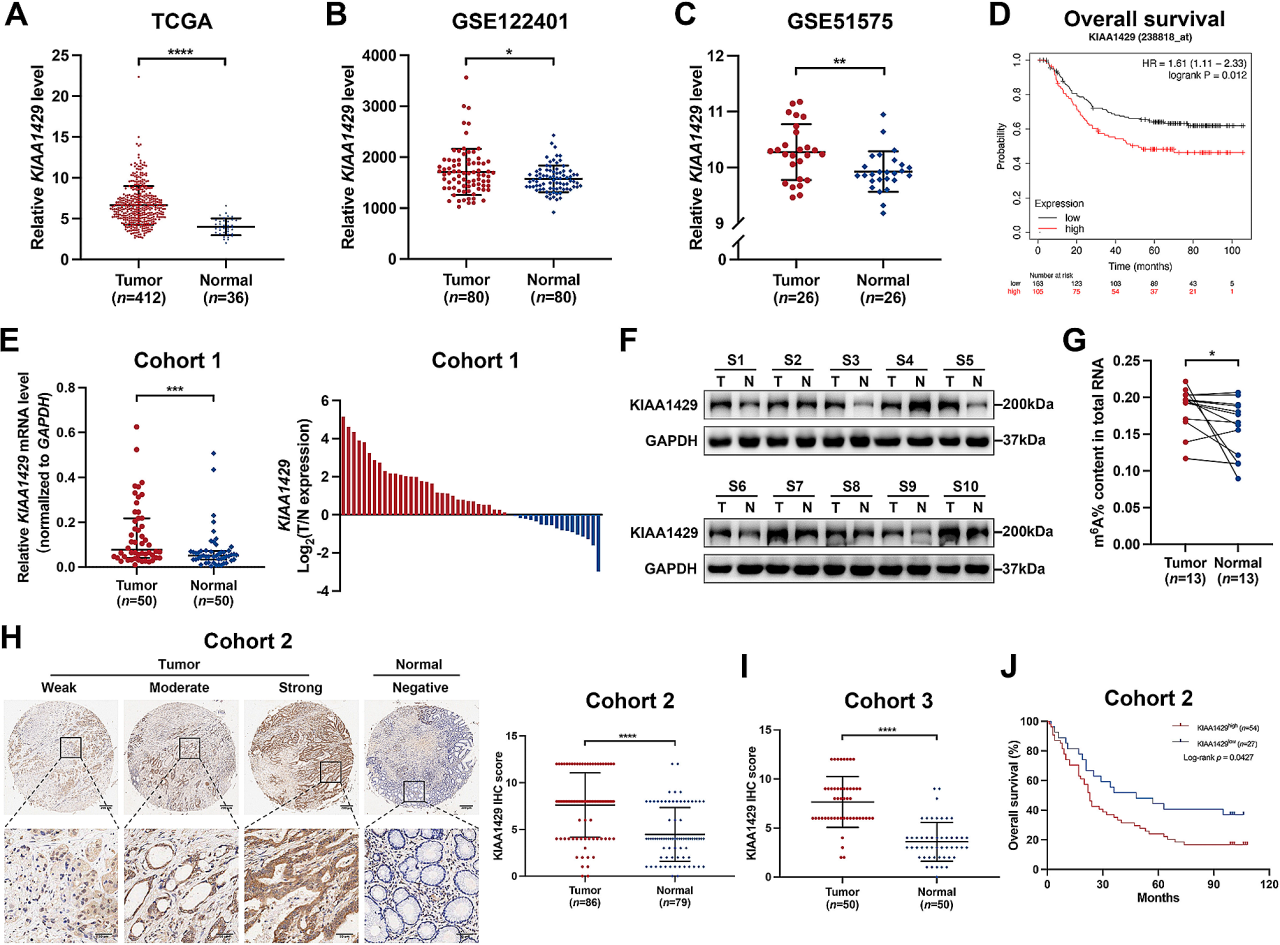
KIAA1429 is upregulated in GC, and this is correlated with poor prognosis. (**A**–**C**) The expression level of KIAA1429 in GC and nontumorous tissue samples in the TCGA-STAD (**A**), GSE122401 (**B**), and GSE51575 (**C**) datasets. (**D**) Kaplan‒Meier analysis of overall survival of GC patients in the GSE62254 dataset in which different mRNA levels of KIAA1429 were analyzed by Kaplan‒Meier plotter. (**E**) Relative mRNA expression of KIAA1429 in 50 paired GC tissues as determined by qRT‒PCR. (**F**) Representative Western blot bands of KIAA1429 in paired GC specimens. (**G**) The m[^6^]A content in the total RNA of 13 paired GC tissues was measured by colorimetric methods. (**H**) Representative IHC images and IHC score of KIAA1429 in cohort 2 (a tissue microarray containing 86 GC tissues and 79 normal gastric tissues). Scale bar: black bar, 200 μm in the overview images; 50 μm in the magnified images. (**I**) IHC scores of KIAA1429 in cohort 3 (50 GC tissues and paired adjacent normal tissues). (**J**) Kaplan‒Meier analysis of overall survival of GC patients with different expression levels of the KIAA1429 protein in cohort 2. The data are presented as the mean ± SD. Two-tailed Student’s *t* test was used for (**A–C, E, G–I**), and the log-rank test was used for (**D** and **J**). *P < 0.05, **P < 0.01, ***P < 0.001, ****P < 0.0001. GC: gastric cancer; TCGA-STAD: The Cancer Genome Atlas-Stomach Adenocarcinoma; T: tumor tissue; N: adjacent normal gastric tissue; IHC: immunohistochemistry

To assess the clinical relevance of KIAA1429 in GC progression, we initially analyzed its expression in GC and nontumorous tissue samples from The Cancer Genome Atlas (TCGA) and Gene Expression Omnibus (GEO) databases. The GSE122401 dataset included 80 early-onset GC tissues and paired normal adjacent tissues, and the GSE51575 dataset comprised 26 pairs of GC tissues and normal adjacent tissues. Our analysis revealed the upregulation of KIAA1429 in GC tissues compared to matched or unmatched normal gastric tissues in the TCGA-Stomach Adenocarcinoma (TCGA-STAD) (Fig. 1A), GSE122401 (Fig. 1B), and GSE51575 (Fig. 1C) datasets. Further Kaplan‒Meier analysis of patients with available clinical data from the GSE62254 dataset indicated that high expression of KIAA1429 in GC tissues tended to be associated with worse OS (Fig. 1D). Moreover, we assessed the expression of KIAA1429 mRNA and protein in paired fresh GC tissues from the First Affiliated Hospital of Zhejiang University using qRT‒PCR and Western blotting. Consistent with the database findings, the expression of KIAA1429 mRNA and protein was significantly greater in GC tissues than in corresponding adjacent normal tissues (Fig. 1E and F). Additionally, the m^6^A content of total RNA was elevated in GC tissues (Fig. 1G). Immunohistochemistry (IHC) analysis of cohort 2 (tissue microarray containing 86 GC tissues and 79 normal gastric tissues) and cohort 3 (50 GC tissues and paired adjacent normal tissues) validated the increased KIAA1429 protein levels in GC tissues (Fig. 1H and I). A chi-squared test further indicated that higher expression of KIAA1429 in GC tissues was associated with advanced TNM staging and tumor depth (Supplementary Table S1). Furthermore, Kaplan‒Meier analysis indicated that GC patients with higher expression of KIAA1429 exhibited poorer OS (Fig. 1J). Additionally, qRT-PCR, Western blot, and immunofluorescence (IF) analyses were performed to assess the expression of KIAA1429 in six GC cell lines (AGS, MGC-803, HGC-27, KATO III, MKN-45, and NCI-N87) and the immortalized normal gastric epithelial cell line GES-1. The results revealed that the expression of KIAA1429 mRNA and protein was significantly elevated in GC cell lines compared with the immortalized normal gastric epithelial cell line GES-1 (Supplementary Fig. S1A and S1B) and was mainly located in the nucleus (Supplementary Fig. S1C). Together, these results suggest a potential oncogenic role of KIAA1429 in GC.

### KIAA1429 promotes GC cell growth in vitro and in vivo

**Fig. 2:**
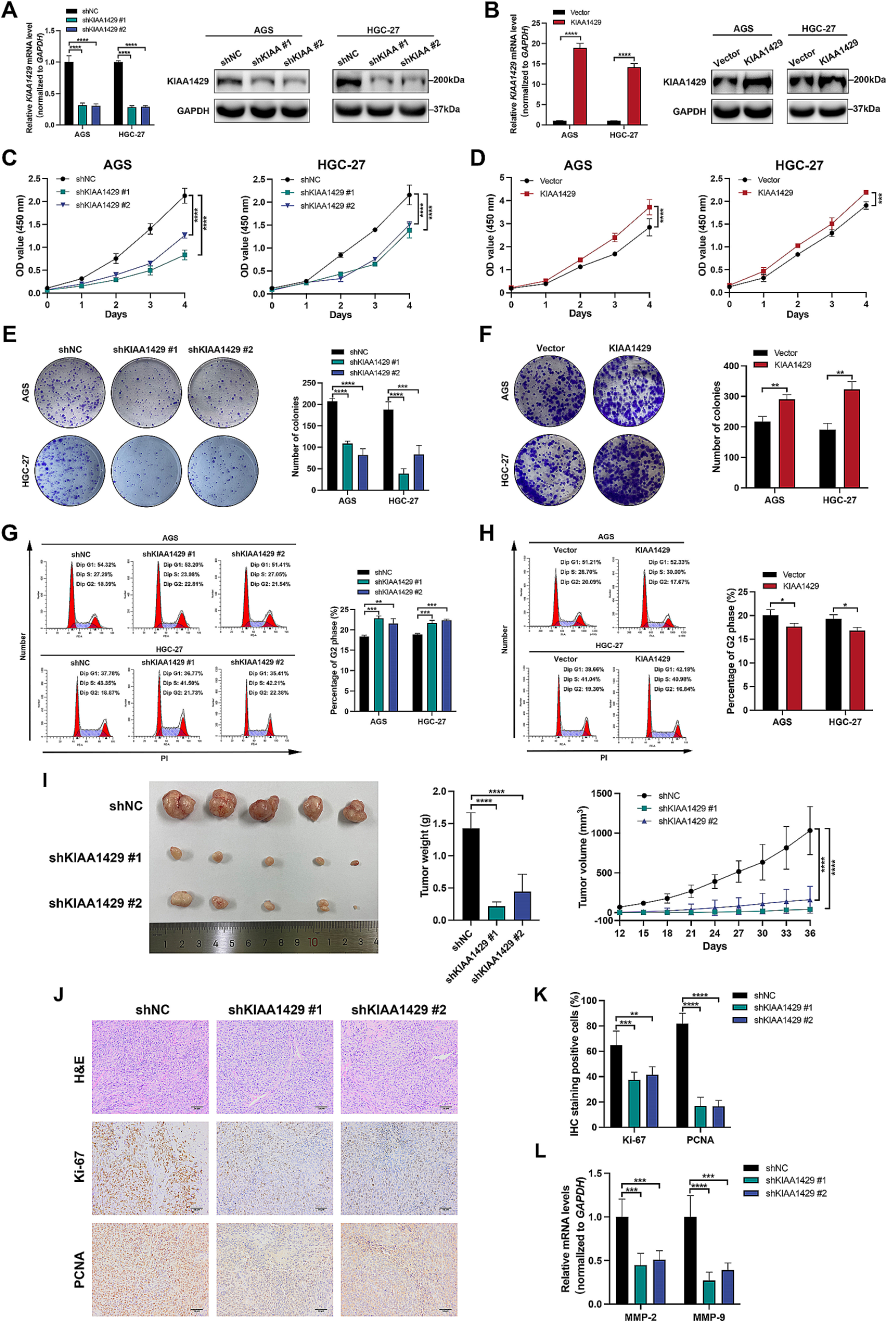
KIAA1429 promotes GC cell growth both in vitro and *in vivo.* (**A–B**) qRT‒PCR and Western blot confirmation of the knockdown (**A**) and overexpression (**B**) efficacy. (**C–D**) Analysis of GC cell proliferation by CCK-8 assays. (**E–F**) Representative results of the colony formation assay. The number of colonies with > 50 cells was calculated. (**G–H**) Flow cytometry analyses of the cell cycle distribution of the indicated GC cells. (**I**) The xenograft models were generated after injection of HGC-27/shNC, HGC-27/shKIAA1429 #1 and HGC-27/shKIAA1429 #2 (*n* = 5/group). Nude mice were euthanized, and the tumors were weighed. The tumor volumes were calculated on the indicated days. (**J**) The tumor sections were subjected to H&E staining or IHC staining using antibodies against Ki-67 and PCNA. (**K**) The percentage of Ki-67- and PCNA-positive cells in subcutaneous tumors. (**L**) Relative mRNA levels of MMP-2 and MMP-9 in subcutaneous tumors. The data are presented as the means ± SDs. Dunnett *t* test for (**A, C, E, G, I, K, L**) and two-tailed Student’s *t* test for (**B, D, F, H**). *P < 0.05, **P < 0.01, ***P < 0.001, ****P < 0.0001. GC: gastric cancer

To elucidate the role of KIAA1429 in GC progression, KIAA1429 was stably silenced and overexpressed in AGS and HGC-27 cell lines using lentivirus. The effectiveness of knockdown and overexpression was confirmed through qRT‒PCR and Western blot analysis (Fig. 2A and B). Subsequent assessments using the Cell Counting Kit-8 (CCK-8) assay, colony formation assay, and ethynyl deoxyuridine (EdU) assay revealed that KIAA1429 knockdown significantly inhibited GC cell growth (Fig. 2C, Supplementary Fig. S2A and S2B) and colony formation efficiency (Fig. 2E). Conversely, overexpression of KIAA1429 significantly increased cell proliferation and colony formation (Fig. 2D and F, Supplementary Fig. S2A and S2B). Flow cytometry analysis of cell apoptosis indicated that KIAA1429 knockdown increased the apoptosis rate of AGS and HGC-27 cells (Supplementary Fig. S2C). Additionally, the distribution of GC cells within different stages of the cell cycle was examined by flow cytometry. KIAA1429 silencing in GC cells led to an increase in the percentage of cells in the G2 peak (Fig. 2G), whereas GC cells overexpressing KIAA1429 showed a decrease in the percentage of cells in the G2 peak (Fig. 2H), suggesting that KIAA1429 promotes the G2/M cell cycle transition in GC cells. Western blot analysis further showed that KIAA1429 knockdown upregulated p21 protein levels and downregulated Cyclin B1 protein levels and CDK1 phosphorylation levels, whereas KIAA1429 overexpression had the opposite effects (Supplementary Fig. S3).

Furthermore, we established a subcutaneous tumor-bearing nude mouse model using HGC-27 cells with stable knockdown of KIAA1429. The results demonstrated that tumors formed by HGC-27 cells with KIAA1429 inhibition grew more slowly than those formed by control cells (Fig. 2I). Additionally, the tumor weight and percentage of Ki-67- and PCNA-positive cells in subcutaneous tumors with KIAA1429 inhibition were significantly lower (Fig. 2J and K). The mRNA expression of MMP-2 and MMP-9 in subcutaneous tumors was also significantly decreased when KIAA1429 was knocked down (Fig. 2L). Collectively, these results indicate that KIAA1429 inhibits GC cell growth in vitro and in vivo by regulating cell cycle progression and cell apoptosis.

### KIAA1429 promotes GC metastasis in vitro and in vivo

**Fig. 3:**
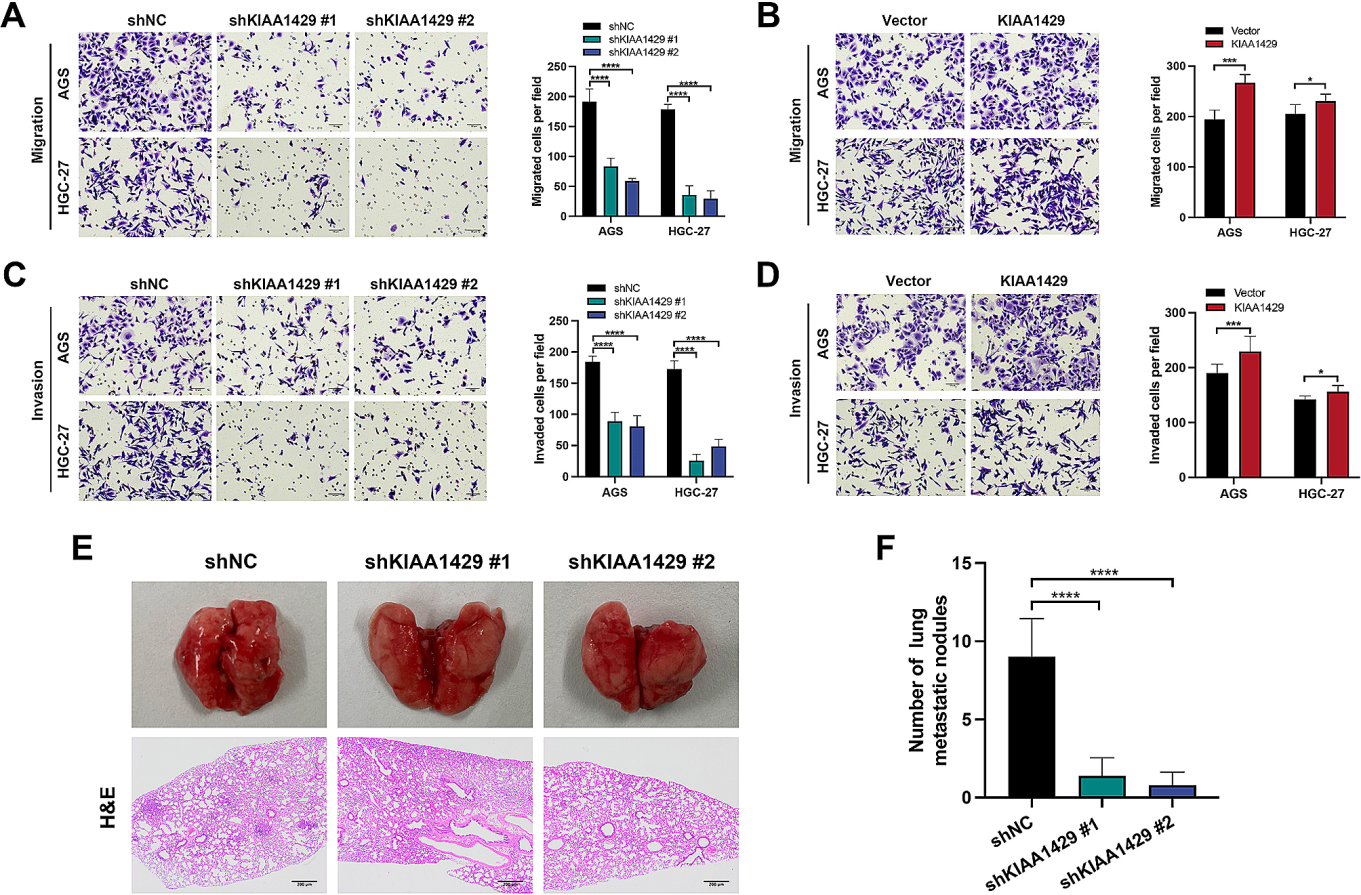
KIAA1429 promotes GC metastasis in vitro and in vivo. (**A–B**) Representative images of migration assays in GC cells with KIAA1429 knockdown (**A**) and overexpression (**B**) (left panel). Scale bar: black bar, 50 μm. The number of migrated cells per field was quantified (right panel). (**C–D**) Representative images of invasion assays for GC cells in the KIAA1429 knockdown (**C**) and overexpression groups (**D**) (left panel). Scale bar: black bar, 50 μm. The number of invaded cells per field was quantified (right panel). (**E**) The experimental lung metastasis models were generated after intravenous injection of MKN-45/shNC, MKN-45/shKIAA1429 #1 or MKN-45/shKIAA1429 #2. Lung tissues were harvested, and lung sections were subjected to H&E staining. Scale bar: black bar, 200 μm. (**F**) The number of lung metastatic nodules. The data are presented as the means ± SDs. Dunnett *t* test for (**A, C, F**) and two-tailed Student’s *t* test for (**B, D**). *P < 0.05, ***P < 0.001, ****P < 0.0001. GC: gastric cancer

Transwell assays were performed to investigate the effects of KIAA1429 on GC cell migration and invasion in vitro. The findings revealed a significant reduction in migration and invasion following KIAA1429 knockdown (Fig. 3A and C). Conversely, the overexpression of KIAA1429 reversed this effect (Fig. 3B and D), suggesting that KIAA1429 promoted GC cell migration and invasion in vitro. We next investigated the effects of KIAA1429 on GC metastasis in vivo using an experimental lung metastasis model. The results showed that KIAA1429 knockdown significantly inhibited the lung metastasis of MKN-45 GC cells (Fig. 3E and F). Together, these results suggest that KIAA1429 has a prometastatic role in GC.

### RASD1 functions as a downstream effector of KIAA1429 in GC

**Fig. 4:**
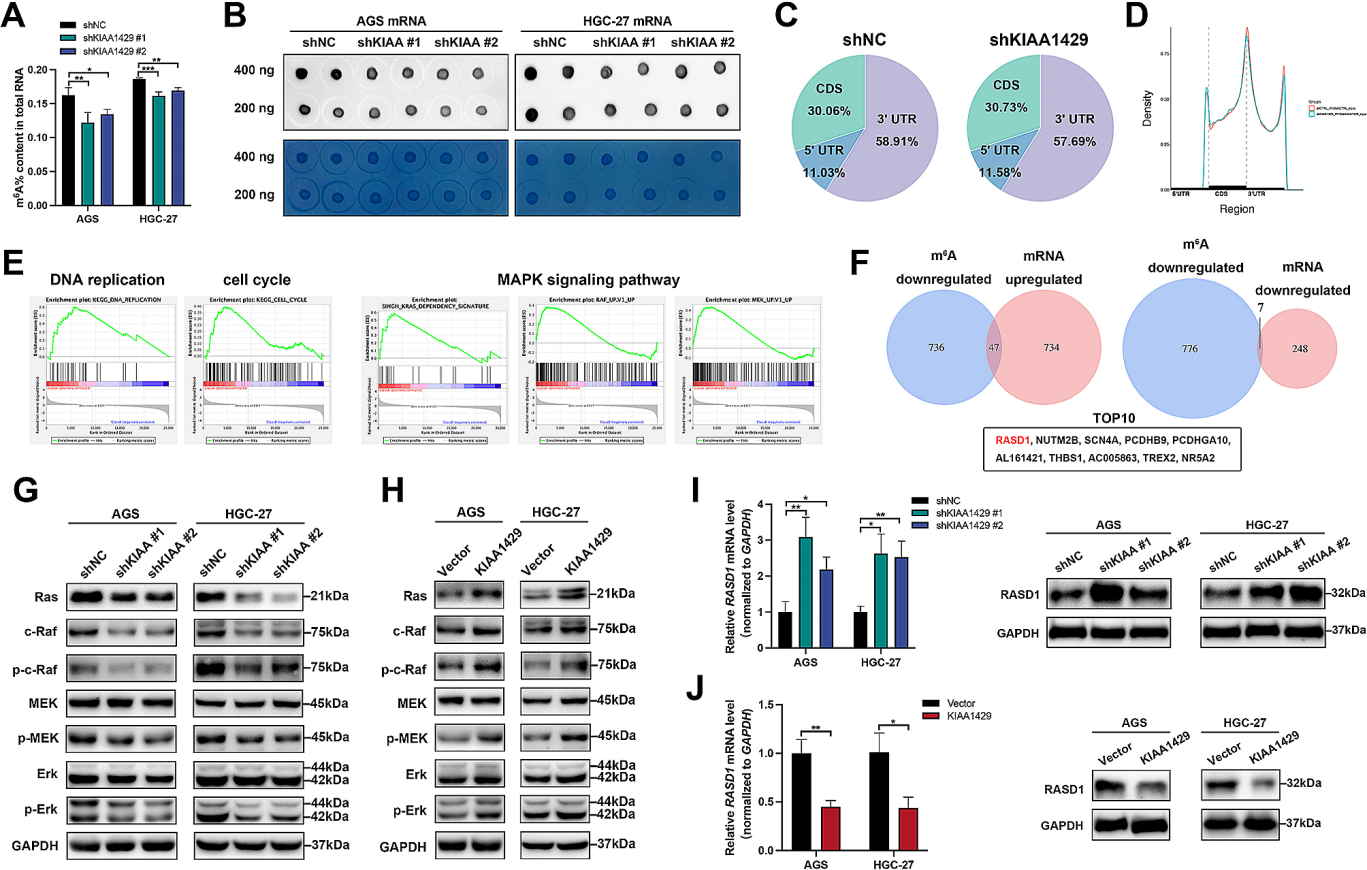
RASD1 is a downstream effector of KIAA1429. (**A**) Colorimetric assay measurement of the m[^6^]A content in total RNA. (**B**) m[^6^]A dot blot analysis of the m[^6^]A content in mRNA. m[^6^]A content (upper panel); RNA loading control (lower panel). (**C**) Pie chart showing the distribution of m[^6^]A modifications on transcripts. (**D**) Distribution density of m[^6^]A modifications on transcripts. (**E**) Enrichment plots from GSEA. (**F**) Venn diagram analyses of genes with reduced m[^6^]A peaks and increased/decreased mRNA levels. (**G–H**) Representative Western blot bands for the MAPK signaling pathway-related proteins after knockdown (**G**) or overexpression (**H**) of KIAA1429. (**I–J**) Relative mRNA expression of RASD1 after knockdown or overexpression of KIAA1429 (left panel). Representative Western blot bands of RASD1 after knockdown or overexpression of KIAA1429 (right panel). The data are presented as the means ± SDs. Dunnett *t* test for (**A, I**) and two-tailed Student’s *t* test for (**J**). *P < 0.05, **P < 0.01, ***P < 0.001. GC: gastric cancer; GSEA: gene set enrichment analysis

To elucidate the underlying mechanism by which KIAA1429 promotes the growth and metastasis of GC, the m^6^A levels in total RNA and mRNA of KIAA1429-knockdown GC cells were assessed using a colorimetric assay and m^6^A dot blot, respectively. The results revealed that silencing KIAA1429 led to a significant reduction in the m^6^A content in both the total RNA and mRNA of GC cells (Fig. 4A and B). Subsequently, MeRIP-seq and RNA-seq analyses were performed to elucidate the landscape of downstream targets influenced by KIAA1429-mediated m^6^A modification in AGS cells following KIAA1429 knockdown. The sequencing results indicated that the majority of the transcripts carried m^6^A marks exclusively within the 3’ UTR, particularly in proximity to the stop codon (Fig. 4C and D). Gene set enrichment analysis (GSEA) revealed a specific enrichment of factors in the mitogen-activated protein kinase/extraneous signal-regulated kinase (MAPK/ERK) signaling pathway, including the KRAS dependency signature, as well as the RAF and MEK-associated signaling pathways (Fig. 4E). A total of 783 genes exhibited downregulated m^6^A peaks on their transcripts upon KIAA1429 knockdown in AGS cells (log_2_(FC) ≤ -1 and *P* value < 0.05), while 1,036 genes were differentially expressed (|log_2_(FC)| ≥ 1 and *P* value < 0.05) (Fig. 4F). Among the differentially expressed genes, 781 were upregulated, and 255 were downregulated. Western blot results further confirmed that KIAA1429 knockdown significantly reduced the protein levels of Ras and c-Raf, as well as the phosphorylation levels of c-Raf, MEK, and Erk, indicating that KIAA1429 positively regulates the MAPK/ERK signaling pathway (Fig. 4G and H).

Considering that KIAA1429 functions as a regulatory subunit of the m^6^A methyltransferase complex [24], we performed Venn diagram analyses to identify genes with reduced m^6^A peaks and altered mRNA levels following KIAA1429 knockdown (Fig. 4F). The top 10 most significantly altered genes included RASD1, NUTM2B, SCN4A, PCDHB9, PCDHGA10, AL161421, THBS1, AC005863, TREX2, and NR5A2 (Fig. 4F). Subsequent qRT‒PCR and Western blot analysis confirmed that KIAA1429 knockdown decreased *RASD1* mRNA and protein expression, while KIAA1429 overexpression had the opposite effect (Fig. 4I and J), suggesting that KIAA1429 positively regulates RASD1 expression in GC cell lines.

### KIAA1429 positively regulates m^6^ A modification of *RASD1* mRNA

**Fig. 5:**
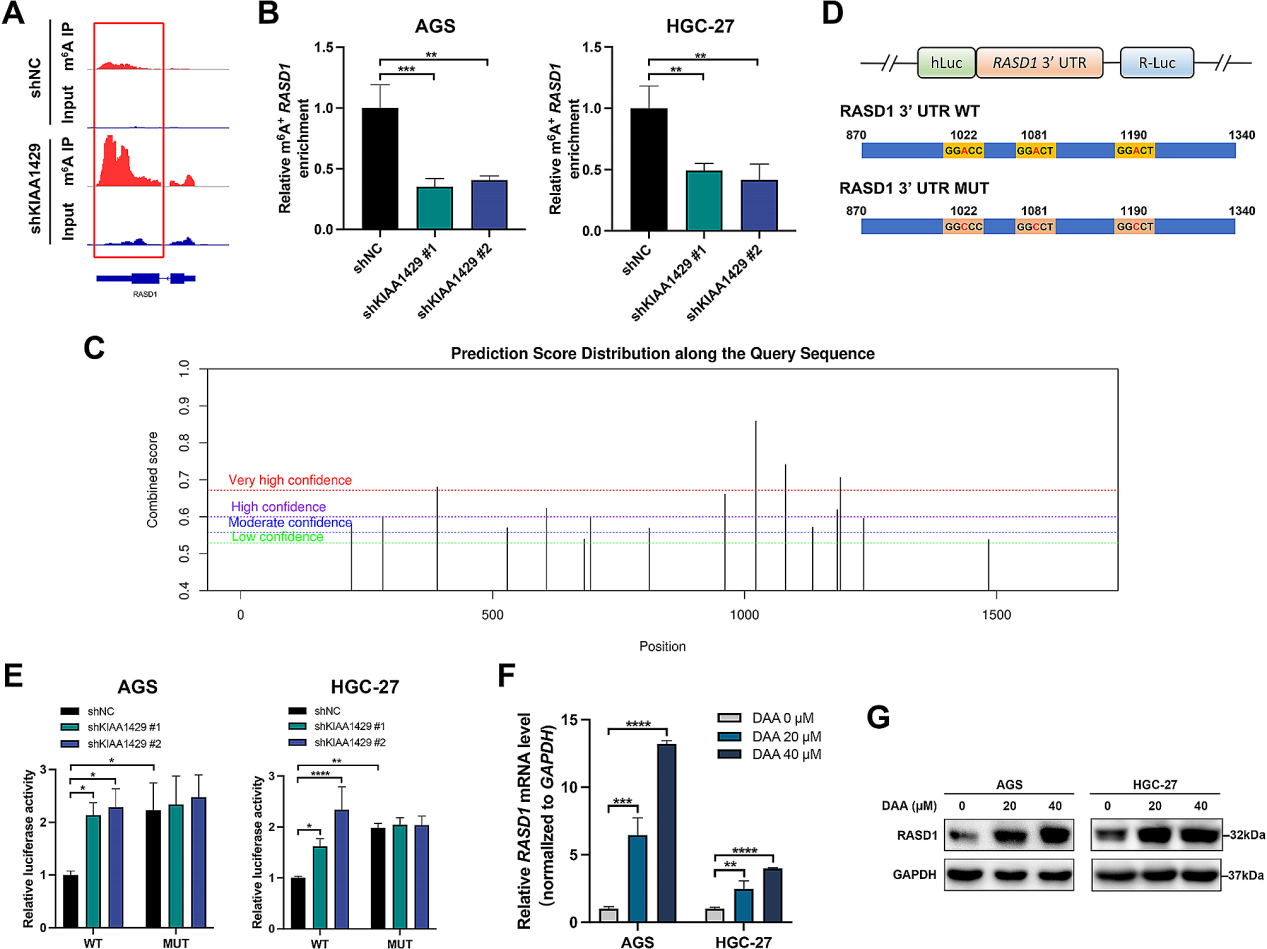
KIAA1429 positively regulates the m[^6^]A modification of *RASD1* mRNA. (**A**) IGV plots showing m[^6^]A abundance on *RASD1* mRNA after KIAA1429 knockdown based on MeRIP-seq data. (**B**) The m[^6^]A level of *RASD1* mRNA was measured by MeRIP-qPCR after KIAA1429 knockdown in GC cells. (**C**) SRAMP prediction of potential m[^6^]A modification sites in RASD1. (**D**) Schematic representation of wild-type (*RASD1* mRNA WT) and mutant (*RASD1* mRNA MUT) *RASD1* mRNA constructs. (**E**) Luciferase activity of *RASD1* mRNA as determined by dual-luciferase assays following KIAA1429 knockdown or in cells with mutant m[^6^]A modification sites. (**F**) Relative mRNA expression of RASD1 in GC cells after DAA treatment. (**G**) Representative Western blot bands showing RASD1 in GC cells after DAA treatment. The data are presented as the means ± SDs. Dunnett *t* test for (**B, E, F**). *P < 0.05, **P < 0.01, ***P < 0.001, ****P < 0.0001. IGV: Integrative Genomics Viewer; GC: gastric cancer; DAA: 3-deazaadenosine

We further explored the specific molecular mechanisms through which KIAA1429 regulates *RASD1* mRNA. First, MeRIP-seq data were visualized using Integrative Genomics Viewer (IGV). As shown in Fig. 5A, m^6^A peaks were mainly distributed at the 3′ UTR of *RASD1* mRNA. After normalization to the input, the m^6^A peak on *RASD1* mRNA in KIAA1429-knockdown GC cells was 0.367-fold lower than that in control cells. MeRIP-qPCR results further demonstrated a significant reduction in m^6^A modification of *RASD1* mRNA upon KIAA1429 knockdown in both AGS and HGC-27 cells (Fig. 5B). Subsequently, we used the SRAMP website to predict m^6^A modification sites on the full-length mRNA sequence of RASD1. The findings revealed the existence of four m^6^A modification sites on the *RASD1* mRNA sequence with very high confidence, three of which were located on the 3′ UTR of *RASD1* mRNA (Fig. 5C). Three specific positions (1022, 1081, and 1190) on the 3′ UTR of *RASD1* mRNA featuring the sequence “GGACC” or “GGACU” were selected for further investigation. We designed RASD1 3′ UTR WT/MUT plasmids, and dual-luciferase reporter assays were subsequently conducted (Fig. 5D and E). KIAA1429 knockdown significantly increased the luciferase activity of *RASD1* mRNA, while this effect was attenuated when the site was mutated (Fig. 5E). Next, GC cells were treated with different concentrations of the methyltransferase inhibitor 3-deazaadenosine (DAA). The results revealed that DAA treatment significantly increased the mRNA and protein expression of RASD1 in a concentration-dependent manner (Fig. 5F and G). In summary, these findings demonstrate that KIAA1429 plays a positive regulatory role in the m^6^A modification of *RASD1* mRNA, with modification sites identified as adenosines at positions 1022, 1081, or 1190.

### KIAA1429 maintains *RASD1* mRNA stability through an m^6^ A-YTHDF2-dependent mechanism

**Fig. 6:**
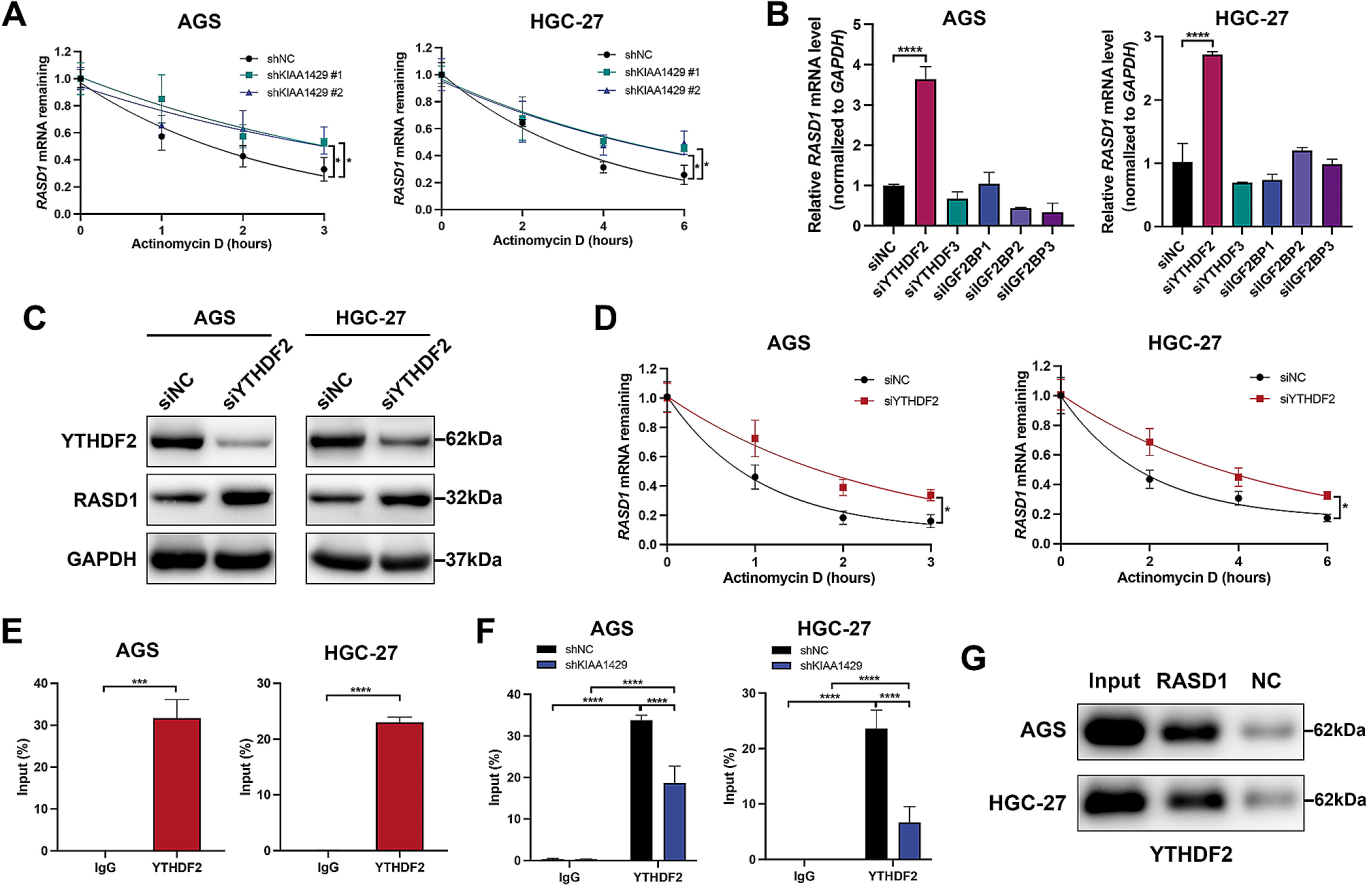
KIAA1429 enhances *RASD1* mRNA stability in an m[^6^]A-YTHDF2-dependent manner. (**A**) *RASD1* mRNA stability at different time points after actinomycin D treatment. (**B**) Relative mRNA expression of RASD1 in GC cells after knockdown of YTHDF2, YTHDF3, IGF2BP1, IGF2BP2, or IGF2BP3. (**C**) Representative Western blot bands of RASD1 in GC cells after YTHDF2 knockdown. (**D**) *RASD1* mRNA stability at different time points after actinomycin D treatment. (**E**) RIP assay showing the interaction between the YTHDF2 protein and *RASD1* mRNA. (**F**) RIP assay illustrating that KIAA1429 knockdown decreased the interaction between the YTHDF2 protein and *RASD1* mRNA. (**G**) RNA pull-down assay was carried out to confirm the interaction between *RASD1* mRNA and the YTHDF2 protein. The data are presented as the means ± SDs. Dunnett *t* test for (**A, B, F**) and two-tailed Student’s *t* test for (**D, E**). *P < 0.05, **P < 0.01, ***P < 0.001, ****P < 0.0001. GC: gastric cancer

Dynamic m^6^A modification plays a pivotal role in various cellular processes, notably promoting mRNA degradation in the cytoplasm [5]. RNA stability assays demonstrated that KIAA1429 knockdown significantly reduced the mRNA stability of RASD1 (Fig. 6A). m^6^A modifications are recognized by reader proteins and are crucial for facilitating the diverse functions of m^6^A in gene regulation. The m^6^A reader proteins implicated in mediating RNA degradation and stability include YTHDF2, YTHDF3, IGF2BP1, IGF2BP2, and IGF2BP3 [25, 26]. Subsequent qRT‒PCR revealed that the knockdown of YTHDF2, as opposed to YTHDF3, IGF2BP1, IGF2BP2, or IGF2BP3, significantly increased the *RASD1* mRNA level (Fig. 6B). Western blot analysis further confirmed that YTHDF2 knockdown increased the protein level of RASD1 (Fig. 6C). YTHDF2 knockdown significantly increased *RASD1* mRNA stability in AGS and HGC-27 cells (Fig. 6D). Additionally, the RIP assay demonstrated a direct interaction between the YTHDF2 protein and *RASD1* mRNA (Fig. 6E), with KIAA1429 knockdown resulting in a decrease in this interaction (Fig. 6F). The interaction between *RASD1* mRNA and YTHDF2 was further determined by RNA pull-down assays, and the results showed that biotin-labeled *RASD1* mRNA exhibited the ability to harbor YTHDF2 protein (Fig. ​6G). Collectively, these findings highlight that KIAA1429 maintains *RASD1* mRNA stability through an m^6^A-YTHDF2-dependent mechanism.

### Downregulation of RASD1 in GC and its impact on GC cell proliferation, migration, and invasion

**Fig. 7:**
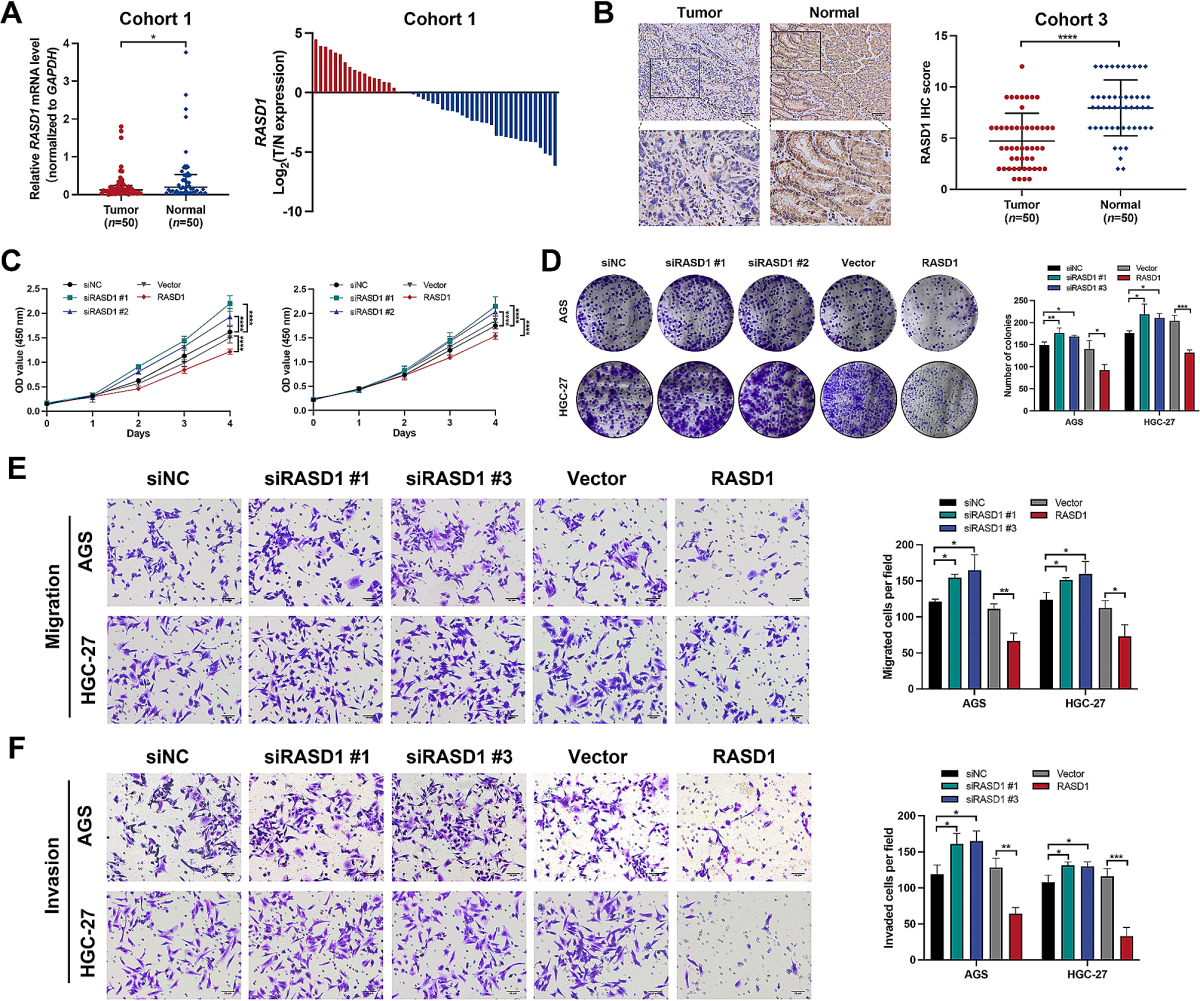
RASD1 is downregulated in GC and inhibits the proliferation, migration, and invasion of GC cells. (**A**) Relative mRNA expression of RASD1 in 50 paired GC tissues analyzed by qRT‒PCR. (**B**) Representative IHC images and IHC scores of KIAA1429 in cohort 3 (50 GC tissues and paired adjacent normal tissues). Representative IHC images of RASD1 (left panel). IHC score of RASD1 (right panel). Scale bar: black bar, 50 μm in the overview images; 20 μm in the magnified images. (**C**) CCK-8 assays for GC cell proliferation. (**D**) Representative results of colony formation assays. The numbers of colonies containing > 50 cells were calculated. (**E**) Representative images of migration assays in RASD1-knockdown and RASD1-overexpressing GC cells (left panel). Scale bar: black bar, 50 μm. Quantification of migrated cells per field (right panel). (**F**) Representative images of the invasion assays in GC cells with KIAA1429 knockdown and overexpression (left panel). Scale bar: black bar, 50 μm. Quantification of invaded cells per field (right panel). The data are presented as the means ± SDs. Dunnett *t* test for (**C–F**) and two-tailed Student’s *t* test for (**A–F**). *P < 0.05, **P < 0.01, ***P < 0.001, ****P < 0.0001. GC: gastric cancer

qRT‒PCR analysis of 50 GC tissues and paired adjacent normal tissues demonstrated a significant reduction in *RASD1* mRNA expression in GC (Fig. 7A). Furthermore, IHC analysis of cohort 3, comprising 50 GC tissues and paired adjacent normal tissues, corroborated a significant decrease in RASD1 protein expression (Fig. 7B). Subsequently, siRNAs targeting RASD1 or pcDNA3.1-RASD1 were used to knockdown or overexpress RASD1, respectively, in AGS and HGC-27 cells. RASD1 knockdown significantly enhanced GC cell proliferation, colony formation ability, migration, and invasion, whereas RASD1 overexpression exerted the opposite effects (Fig. 7C–F). Collectively, these findings suggest a potential tumor-suppressive role for RASD1 in GC.

### RASD1 mediates the pro‑oncogenic role of KIAA1429

**Fig. 8:**
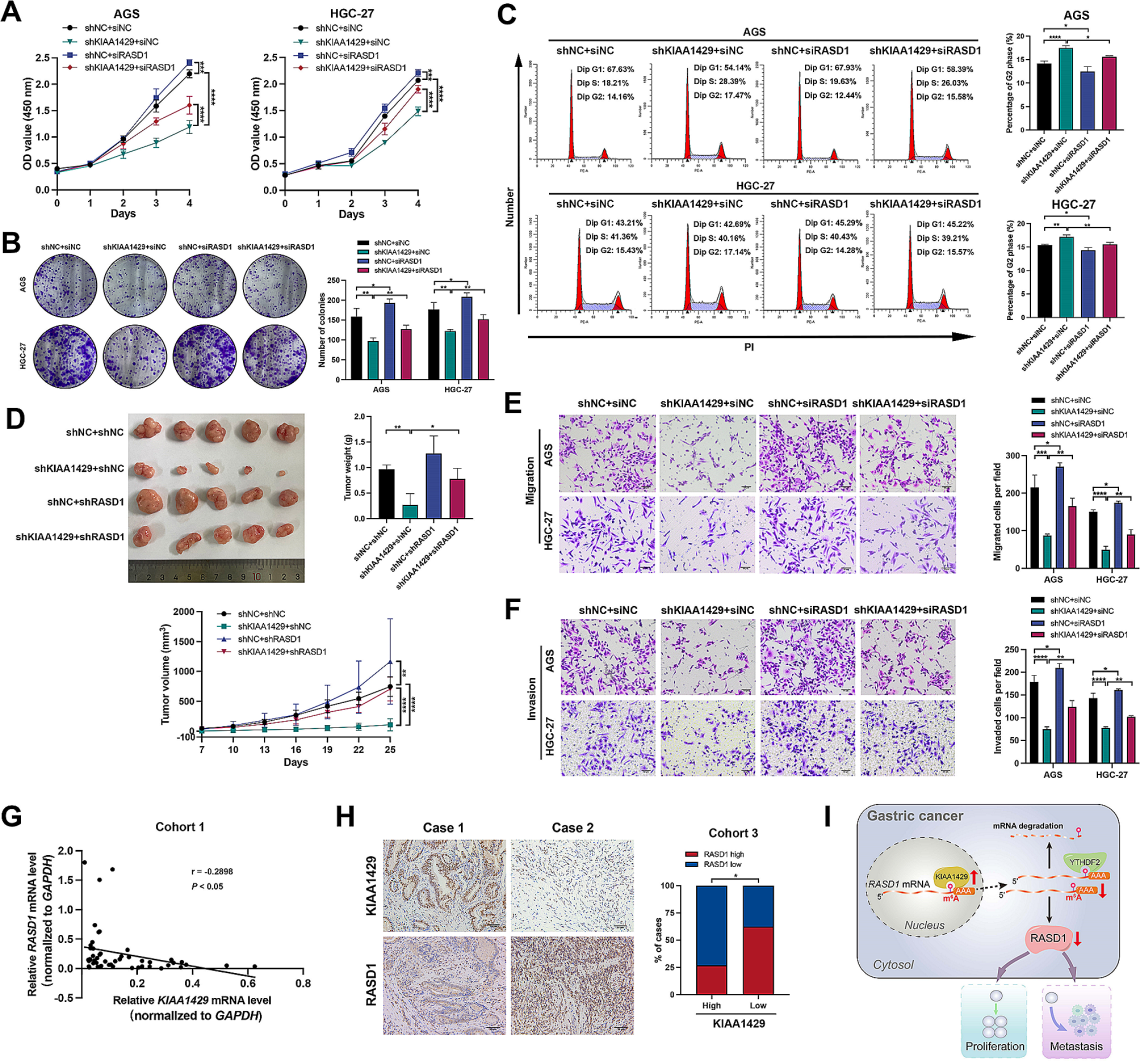
RASD1 mediates the pro‑oncogenic role of KIAA1429, and the expression of RASD1 is negatively correlated with KIAA1429 expression in GC. (**A**) CCK-8 assay analysis of GC cell proliferation. (**B**3) Representative results of colony formation assays. The numbers of colonies containing > 50 cells were calculated. (**C**) Flow cytometry analyses of the cell cycle distribution of the indicated GC cells. (**D**) Xenograft models were generated after injection of HGC-27/shNC + shNC, HGC-27/shKIAA1429 + shNC, HGC-27/shNC + shRASD1, or HGC-27/ shKIAA1429 + shNC (*n* = 5/group). Nude mice were euthanized, and then the tumors were weighed. The tumor volumes were measured on the indicated days. (**E**) Representative images of GC cell migration (left panel). Scale bar: black bar, 50 μm. The number of migrated cells per field was quantified (right panel). (**F**) Representative images of the invasion assay in GC cells (left panel). Scale bar: black bar, 50 μm. The number of migrated cells per field was quantified (right panel). (**G**) qRT‒PCR analysis of the correlation between the mRNA levels of KIAA1429 and RASD1 in 50 GC specimens. (**H**) Representative IHC images of KIAA1429 and RASD1 in 2 patients (left panel). Scale bar: black bar, 50 μm. Percentage of high/low RASD1 protein expression in GC tissues with high or decreased KIAA1429 expression (right panel). (**I**) A diagram illustrating the mechanisms by which KIAA1429 promotes GC progression by destabilizing *RASD1* mRNA in an m[^6^]A-YTHDF2-dependent manner. The data are presented as the means ± SDs. Dunnett *t* test for (**A–F**), Pearson correlation analysis for H, and chi-squared test for I. *P < 0.05, **P < 0.01, ***P < 0.001, ****P < 0.0001. GC: gastric cancer

To further elucidate the relationship between KIAA1429 and RASD1, we next performed rescue experiments. CCK-8, colony formation, and flow cytometry analyses revealed that GC cell proliferation and colony formation were reduced and that the increase in the percentage of cells in the G2 phase was partially reversed after transfection with RASD1 siRNA (Fig. 8A–C). Subcutaneous xenograft assays in nude mice demonstrated that reduced tumor growth and decreased tumor weight were also partially reversed after RASD1 knockdown (Fig. 8D). Transwell assay results indicated that RASD1 knockdown partially rescued the shKIAA1429-induced impairment of migration and invasion ability in AGS and HGC-27 cells (Fig. 8E and F). These results collectively establish that RASD1 mediates the pro‑oncogenic role of KIAA1429.

### Negative correlation between RASD1 and KIAA1429 expression in GC

We next investigated the relationship between the expression levels of KIAA1429 and RASD1 in GC. qRT‒PCR analysis of 50 GC specimens revealed a negative association between the expression of KIAA1429 and RASD1 (Fig. 8G). IHC staining for KIAA1429 and RASD1 in cohort 3 further validated this correlation through chi-squared analysis (Fig. 8H).

## Discussion

In this study, we observed that KIAA1429 is upregulated in GC and is associated with a poor prognosis. Through in vitro experiments, we demonstrated that KIAA1429 plays a crucial role in facilitating GC cell proliferation, G2/M phase cell cycle transition, migration, and invasion. Moreover, in vivo studies revealed that KIAA1429 knockdown impedes GC growth and suppresses its metastatic capacity. Mechanistically, KIAA1429 was found to enhance the m^6^A modification of *RASD1* mRNA, resulting in a reduction in the YTHDF2-mediated stability of *RASD1* mRNA, ultimately leading to an increase in RASD1 expression.

The role of KIAA1429 in various cancers has been extensively investigated in previous studies. The upregulation of KIAA1429 has been reported in liver cancer [19, 27], colorectal cancer [21, 28], breast cancer [22, 29], lung cancer [17, 30], diffuse large B-cell lymphoma [31], and Ewing sarcoma [32]. Additionally, KIAA1429 has been implicated in promoting the epithelial–mesenchymal transition (EMT) process in breast cancer [33] and enhancing chemoresistance in lung cancer [18, 34]. In GC, Miao et al. reported elevated expression of KIAA1429 in GC tissues, linking its upregulation to enhanced GC growth both in vitro and in vivo [23]. Furthermore, KIAA1429 has been shown to contribute to cisplatin resistance [10] and oxaliplatin resistance [35] in GC cells. Our study aligns with these findings, demonstrating upregulated mRNA and protein expression of KIAA1429 in GC tissues from the TCGA database, GEO database, and clinical specimens. Notably, higher expression of KIAA1429 was associated with a poor prognosis, consistent with previous research findings [23]. Additionally, our results reveal the pro-oncogenic nature of KIAA1429, which promotes GC cell proliferation, G2/M cell cycle transition, migration and invasion in vitro. However, a previous study reported that KIAA1429 knockdown led to S phase cell cycle arrest in SGC-7901 and MGC-803 GC cells [23], which is inconsistent with our findings. We hypothesize that such discrepancies may stem from variations in the cell lines used. The findings from our in vivo experiments further corroborate that KIAA1429 knockdown inhibits GC growth and metastatic capacity. Therefore, our findings present compelling evidence for the pro-oncogenic role of KIAA1429 in GC.

KIAA1429 is the largest known protein of the m^6^A methyltransferase complex that can bind RNA [15]. The regulatory mechanisms of KIAA1429 in various malignancies can be categorized into two groups: those dependent on m^6^A modification[17–19,28−31] and those independent of m^6^A modification [21–23, 33]. As a “writer” of m^6^A modification, KIAA1429 can modulate the m^6^A modification of mRNAs of tumor-promoting genes or tumor suppressor genes, thereby altering their metabolism, function and expression. KIAA1429 also affects the survival, proliferation, self-renewal, differentiation and invasion of cancer cells [36]. Previous studies have reported that KIAA1429 regulates *c-Jun* mRNA in a non-m^6^A-dependent manner in GC [23]. In our study, we first analyzed changes in the m^6^A modification levels of total RNA and mRNA in GC cells after KIAA1429 knockdown. The results showed that the m^6^A levels of total RNA and mRNA decreased after KIAA1429 knockdown in GC cells, suggesting that KIAA1429 can promote GC progression by modulating the m^6^A modification of downstream target mRNAs. In addition, MeRIP-seq combined with RNA-seq revealed that RASD1 may be a potential downstream target of KIAA1429. Furthermore, qRT‒PCR, Western blotting, MeRIP‒qPCR and dual-luciferase reporter gene assays confirmed that KIAA1429 upregulated the m^6^A modification on *RASD1* mRNA and downregulated its expression in GC cells, revealing a novel mechanism through which KIAA1429 promotes GC progression.

m^6^A affects almost all aspects of RNA metabolism, including RNA stability, secondary structure, RNA translation and nuclear export, with a predominant impact on mRNA stability [5]. In this study, the results of the RNA stability assay indicated that *RASD1* mRNA stability was elevated following KIAA1429 knockdown. The biological function of m^6^A modifications on RNA requires recognition by “readers”, which alter RNA metabolic functions [37]. We used siRNAs to knockdown “readers” that were previously found to regulate mRNA stability or degradation [25, 26]. YTHDF2 knockdown significantly upregulated the mRNA and protein expression of RASD1 and enhanced its stability. YTHDF2 is the most abundant “reader” in the YTH family and is known to promote the degradation of mRNA in the cytoplasm [38]. Subsequently, we conducted RIP and RNA pull-down assays to verify the interaction between *RASD1* mRNA and the YTHDF2 protein. We found that the interaction was significantly weakened following KIAA1429 knockdown. Therefore, we concluded that KIAA1429 plays a pro-oncogenic role in GC by upregulating the m^6^A modification of *RASD1* mRNA and suppressing its expression via a YTHDF2-mediated reduction in its mRNA stability.

RASD1, also known as Dexras1, is a member of the RAS superfamily of small G proteins and is known to inhibit the MAPK/ERK signaling cascade [39]. It is widely expressed in various tissues and participates in multiple cellular processes, including cell proliferation, lipid synthesis, and neuronal signaling [40–42]. Aberrant expression of RASD1 has been observed in several malignancies, including osteosarcoma [43], prostate cancer [44], renal cell carcinoma [45], and glioma [46]. Moreover, it has been demonstrated that RASD1 can inhibit the proliferation and induce the apoptosis of breast cancer cells [40, 47], lung cancer cells [40], and prostate cancer cells [44] and suppress the migration and invasion of glioma cells [46]. In addition, RASD1 modulates the development of dexamethasone resistance in multiple myeloma [48]. However, its expression level and mechanisms in GC have been largely unexplored. In this study, we found that RASD1 functions as a tumor suppressor in GC. Notably, the expression of RASD1 was downregulated in GC and inhibited the proliferation, migration, and invasion of GC cells in vitro. Furthermore, the results of rescue experiments showed that RASD1 is a downstream target of KIAA1429, which mediates its pro-oncogenic effects.

This study has several limitations. First, as a regulatory subunit of the m^6^A methyltransferase complex, we did not determine how KIAA1429 recruits the catalytic core complex (METTL3-METTL14) to regioselectively modulate the m^6^A modification of the *RASD1* mRNA 3′ UTR. Further investigations are needed to clarify this phenomenon. In addition, we explored the role and mechanism of KIAA1429 in GC progression. Thus, its role in different stages of GC development remains to be investigated. Furthermore, our results indicate that KIAA1429 has a pro-oncogenic effect on GC cell lines and nude mice. More studies are needed to further confirm this conclusion in other models, including organoids, MNU-induced gastric carcinogenesis models, and transgenic insulin-gastrin mice (INS-GAS mice).

## Conclusions

We have demonstrated that KIAA1429 is upregulated in GC and is correlated with poor patient prognosis. Moreover, KIAA1429 enhances the proliferation and metastasis of GC cells by downregulating the expression of RASD1, a tumor suppressor involved in the MAPK/ERK signaling pathway. Mechanistically, KIAA1429 exerts these effects by destabilizing *RASD1* mRNA in an m^6^A-YTHDF2-dependent manner (Fig. 8I). Therefore, KIAA1429 is a promising prognostic biomarker and an attractive candidate for GC treatment.

### Electronic supplementary material

Below is the link to the electronic supplementary material.

Supplementary Material 1

## Data Availability

The datasets used and/or analyzed during the current study are available from the corresponding author on reasonable request.
