# Supplementary material for: KIAA1429 promotes gastric cancer progression by destabilizing RASD1 mRNA in an m6A-YTHDF2-dependent manner
Source: J Transl Med. 2024 Jun 20;22:584. doi: 10.1186/s12967-024-05375-5 (PMC11191263; doi:10.1186/s12967-024-05375-5)
Supplement: Supplementary file 1 — Supplementary Material 1 [file 12967_2024_5375_MOESM1_ESM.docx]

**Supplementary materials**

**KIAA1429 promotes gastric cancer progression by destabilizing *RASD1* mRNA in an m^6^A-YTHDF2-dependent manner**

**Running title:** Ren et al. KIAA1429 PROMOTES GC THROUGH RASD1

Mengting Ren^1^, Hanghai Pan^1^, Xinxin Zhou^2^, Mosang Yu^2^, Feng Ji^2*^

1 Cancer Center, Department of Gastroenterology, Zhejiang Provincial People's Hospital (Affiliated People's Hospital), Hangzhou Medical College, Hangzhou, Zhejiang, China

2 Department of Gastroenterology, The First Affiliated Hospital, Zhejiang University School of Medicine, Hangzhou, Zhejiang, China

***Correspondence author**

Feng Ji, MD

Department of Gastroenterology

The First Affiliated Hospital, Zhejiang University School of Medicine

79 Qingchun Road, Hangzhou, Zhejiang, 310003, China

Email: jifeng@zju.edu.cn

**Supplementary materials and methods**

**Bioinformatic analysis and** **m^6^A prediction**

Gene expression profiling and clinical data for GC patients were obtained from The Cancer Genome Atlas (TCGA) (https://www.cancer.gov/tcga/) and Gene Expression Omnibus (GEO) (http://www.ncbi.nlm.nih.gov/geo/) databases. Kaplan-Meier plotter (http://kmplot.com/analysis/) was utilized to assess the potential correlation between KIAA1429 mRNA expression and overall survival (OS) in GC patients. Gene set enrichment analysis (GSEA) was performed using GSEA software 4.1.0 (http://www.broadinstitute.org/gsea/). Potential m^6^A sites on *RASD1* mRNA were predicted using the SRAMP online tool (http://www.cuilab.cn/sramp/).

**Total RNA extraction and** **quantitative real-time PCR (qRT****‒PCR)**

Total RNA was extracted from GC cells and tissues using RNAex Pro reagent (Accurate Biology, Changsha, China), followed by reverse transcription into cDNA using the Evo M-MLV reverse transcription kit, according to the manufacturer's protocols (Accurate Biology). qRT**‒**PCR was performed on an Applied Biosystems QuantStudio 5 Real-Time PCR System (ABI, Foster City, CA, USA) using the SYBR Green Premix Pro Taq HS qPCR Kit (Accurate Biology). The data were collected in triplicate and analyzed using the comparative 2^−ΔΔCt^ method. The primers used in this study are listed in Supplementary Table S2.

**Protein extraction and Western blot analysis**

Proteins were extracted from cells or tissues using RIPA buffer (Beyotime, Shanghai, China) containing protease and phosphatase inhibitors (Dawen Biotech, Hangzhou, China). Protein concentrations were determined using the BCA protein assay (Beyotime). Denatured proteins were separated on SDS‒PAGE gels and transferred to polyvinylidene fluoride (PVDF) membranes (Millipore, Billerica, MA, USA). Following a 1-hour block with 5% skim milk at room temperature, the membranes were incubated with primary antibodies overnight at 4 °C. Subsequently, the membranes were incubated with HRP-conjugated secondary antibodies (Solarbio, Beijing, China) for 1 h at room temperature. Protein bands were visualized using enhanced chemiluminescence (ECL) reagent (FUDE, Hangzhou, China) and a chemiluminescent imager. The following primary antibodies were used: anti-KIAA1429 (1∶1,000, Cell Signaling Technology, Danvers, MA, USA), anti-GAPDH (1∶1,000, Cell Signaling Technology), anti-p21 (1∶1,000, Cell Signaling Technology), anti-CDK1 (1∶1,000, Cell Signaling Technology), anti-p-CDK1 (1∶1,000, Cell Signaling Technology), anti-Cyclin B1 (1∶1,000, Cell Signaling Technology), anti-Ras (1∶1,000, Cell Signaling Technology), anti-c-Raf (1∶1,000, Cell Signaling Technology), anti-MEK (1∶1,000, Cell Signaling Technology), anti-p-MEK (1∶1,000, Cell Signaling Technology), anti-Erk (1∶1,000, Cell Signaling Technology), anti-p-Erk (1∶1,000, Cell Signaling Technology), anti-RASD1 (1∶1,000, Abcam, Cambridge, UK), and anti-YTHDF2 (1∶1,000, Abcam).

**Immunohistochemistry (IHC)**

For immunohistochemistry, tumor tissues were fixed with 4% paraformaldehyde, embedded in paraffin, and cut into 5-μm-thick sections. The sections were incubated with primary antibodies overnight at 4 °C, followed by incubation with a secondary antibody (goat anti-rabbit HRP-conjugated antibody; ZSGB-BIO, Beijing, China) for 1 h at 37 °C. The hybridized signals were visualized using diaminobenzidine (DAB; ZSGB-BIO), followed by counterstaining with hematoxylin. The primary antibodies used for IHC were as follows: anti-KIAA1429 (1∶200, Novus, Centennial, CO, USA), anti-RASD1 (1∶100, Abcam), anti-Ki-67(1∶400, Cell Signaling Technology), and anti-PCNA (1∶5,000, Cell Signaling Technology). The IHC staining score was semiquantitatively determined by multiplying the staining intensity and positive rate scores. Staining intensity was scored as 0 (negative), 1 (weak), 2 (moderate), or 3 (strong). The percentage of positive cells was scored as 0 (negative), 1 (1%–25%), 2 (26%–50%), 3 (51%–75%), or 4 (76%–100%). An IHC score ≥8 was classified as high KIAA1429 expression, and a score <8 was classified as low KIAA1429 expression. An IHC score ≥6 was classified as high RASD1 expression, and a score <6 was classified as low RASD1 expression.

**Immunofluorescence (IF)**

GC cells were seeded onto coverslips and cultured overnight. Subsequently, cells were fixed with 4% paraformaldehyde and permeabilized with 0.5% Triton X‐100. The primary antibody (anti-KIAA1429, 1∶150, Novus) was added and incubated overnight at 4 °C, followed by incubation with a secondary antibody (Alexa Fluor 488 goat anti-rabbit antibody; Thermo Fisher Scientific, Waltham, MA, USA) at room temperature for 1 h. Nuclei were counterstained with DAPI (Solarbio), and immunofluorescence images were acquired using an FV3000 confocal microscope (Olympus, Tokyo, Japan).

**Cell Counting Kit-8 (CCK-8) assay**

Cell proliferation was assessed using the Cell Counting Kit-8 (CCK-8; Dojindo, Kumamoto, Japan) following the manufacturer's instructions. Cells were seeded into a 96-well plate at a density of 2,000 cells per well. After culturing for 1, 2, 3, or 4 days, the culture medium in each well was replaced with 100 μL of fresh serum-free medium containing 10 μL of CCK-8 solution. The absorbance at 450 nm was measured using a microplate reader after incubation at 37 °C for 2 h.

**Colony formation assay**

GC cells were seeded into 6-well plates (1,000 cells per well) and cultured for 10 days, and the medium was replaced every other day. Subsequently, colonies were ﬁxed using 4% paraformaldehyde and stained with 0.1% crystal violet. Colonies containing > 50 cells were manually counted and imaged.

**Ethynyl deoxyuridine (EdU) assay**

EdU assays were performed using an EdU kit (Beyotime) following the manufacturer's instructions. GC cells were seeded into 12-well plates and cultured for 24 h, followed by incubation with 10 µM EdU at 37 °C for 2 h. After fixation with 4% paraformaldehyde, the cells were treated with the Click reaction cocktail and Hoechst 33342. Images were captured using an IX71 fluorescence microscope (Olympus).

**Cell apoptosis and cell cycle assays**

Cell apoptosis and cell cycle assays were performed using a FACSCanto II flow cytometer, as previously described^1^. For the cell apoptosis assay, GC cells were collected, and apoptosis was detected using an Annexin V-APC/7-AAD apoptosis kit (MultiSciences, Hangzhou, China) following the manufacturer's instructions. For the cell cycle assay, GC cells were collected, ﬁxed in 75% cold ethanol at -20 °C overnight, and then washed twice with cold PBS. Cell cycle analysis was conducted using a cell cycle detection kit (MultiSciences) according to the manufacturer's instructions. The percentage of apoptotic cells was determined using FlowJo version 10.4.0 software (FlowJo, Ashland, OR, USA), and the cell cycle distribution was determined using ModFit LT 6.0 software (Verity, Topsham, ME, USA).

**Cell migration and invasion assays**

Cell migration and invasion assays were performed using 24-well plates with 8-μm pore size transwell inserts (Corning, NY, USA). For invasion assays, transwell inserts were precoated with Matrigel (1∶5 dilution, Corning). Cells (5×10^4^ for the migration assay and 1×10^5^ for the invasion assay) were seeded into the upper chamber. After 24 h of incubation, migrated or invaded cells were ﬁxed with 4% paraformaldehyde and stained with 0.1% crystal violet. Five random fields of view were photographed under an inverted microscope (Olympus), and the number of stained cells was quantified using ImageJ version 1.52a software (Rawak, Stuttgart, Germany).

**
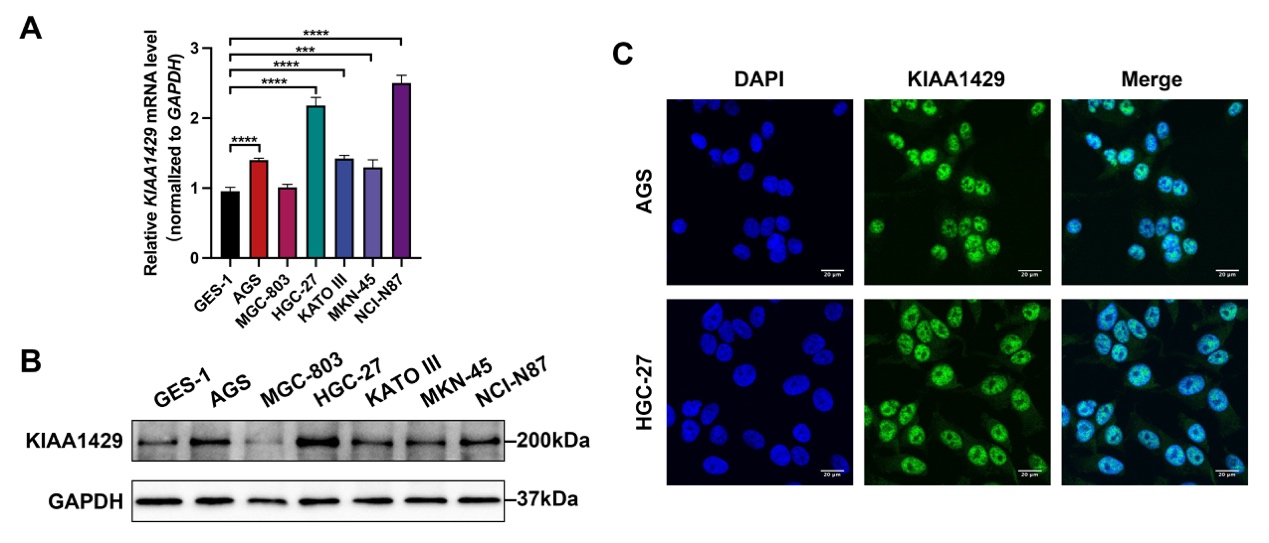
**

**Supplementary Fig. S1** KIAA1429 is upregulated in GC cell lines compared with the immortalized normal gastric epithelial cell line. (A) Relative mRNA expression of KIAA1429 in 6 GC cell lines (AGS, MGC-803, HGC-27, KATO III, MKN-45, and NCI-N87) and the immortalized normal gastric epithelial cell line GES-1. (B) Representative Western blot bands of KIAA1429 in GC cell lines and the GES-1 cell line. (C) Representative IF images of KIAA1429 (green) and DAPI (blue) in AGS and HGC-27 cell lines. Scale bar: white bar, 20 μm. The data are shown as means ± SDs. Dunnett *t* test. ****P* < 0.001, *****P* < 0.0001. GC: gastric cancer; IF: immunofluorescence.


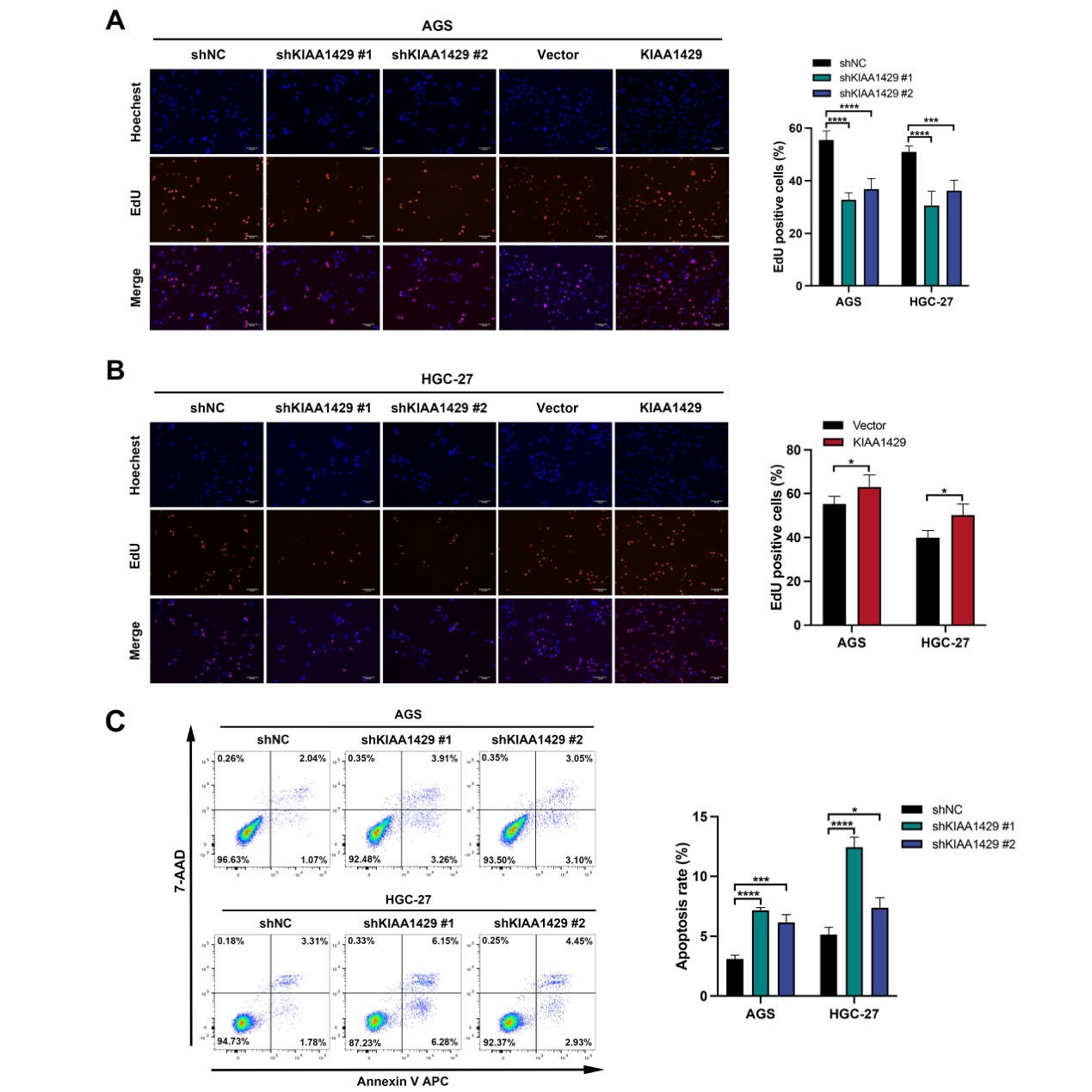


**Supplementary Fig. S2** KIAA1429 promotes GC cell proliferation, and knockdown of KIAA1429 induces GC cell apoptosis. (A–B) Representative EdU fluorescence images and percentages of EdU-positive cells. Scale bar: white bar, 50 μm. (C) GC cell apoptosis assessed by ﬂow cytometry. Annexin Ⅴ-positive/7-AAD-negative (right lower quadrant) cells and Annexin Ⅴ-positive/7-AAD-positive (right upper quadrant) cells were analyzed for the percentage of apoptotic cells. The data are shown as means ± SDs. Dunnett’s *t* test for (A, C) and two-tailed Student's *t* test for (B). **P* < 0.05, ****P* < 0.001, *****P* < 0.0001. GC: gastric cancer.


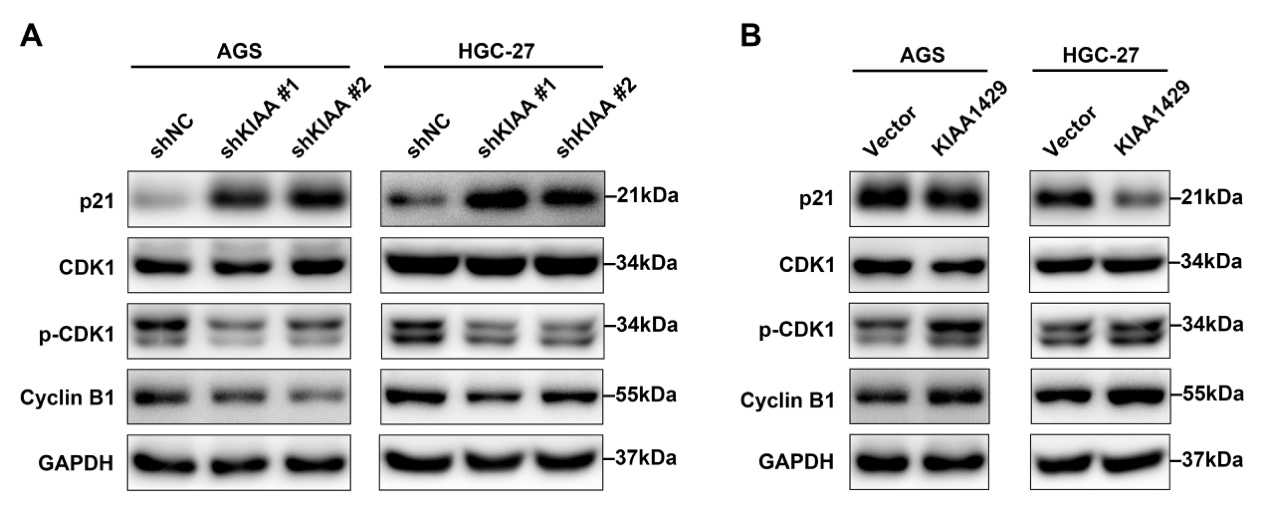


**Supplementary Fig. S3** KIAA1429 alters the expression of cell cycle regulators involved in the G2/M transition. (A) Representative Western blot bands of cell cycle regulators of the G2/M transition after knockdown of KIAA1429. (B) Representative Western blot bands of cell cycle regulators of the G2/M transition after overexpression of KIAA1429.

| **Supplementary Table S1** Correlation between KIAA1429 expression level and clinicopathologic characteristics of GC patients. | | | | | |
| --- | --- | --- | --- | --- | --- |
| Variables | KIAA1429 expression | | *χ*2 | *P* value |  |
|  | Low | High |  |  |  |
| Age (year) |  |  |  |  |  |
| ≤60 | 7 (25%) | 21 (75%) | 0.788 | 0.375 |  |
| ＞60 | 20(34.5%) | 38 (65.5%) |  |  |  |
| Gender |  |  |  |  |  |
| Male | 21 (37.5%) | 35 (62.5%) | 2.778 | 0.096 |  |
| Female | 6 (20.0%) | 24 (80.0%) |  |  |  |
| Grade of differentiation |  |  |  |  |  |
| Low | 15 (32.6%) | 31 (67.4%) | 0.068 | 0.795 |  |
| Medium | 12 (30.0%) | 28 (70.0%) |  |  |  |
| TNM classification |  |  |  |  |  |
| Ⅰ–Ⅱ | 10 (52.6%) | 9 (47.4%) | 5.106 | **0.024*** |  |
| Ⅲ–IV | 17 (25.4%) | 50 (74.6%) |  |  |  |
| Tumor depth |  |  |  |  |  |
| T1–T2 | 8 (66.7%) | 4 (33.3%) | 6.264 | **0.012*** |  |
| T3–T4 | 19 (25.7%) | 55 (74.3%) |  |  |  |
| Lymph node metastasis |  |  |  |  |  |
| Negative | 9 (40.9%) | 13 (59.1%) | 1.242 | 0.265 |  |
| Positive | 18 (28.1%) | 46 (71.9%) |  |  |  |
| Distant metastasis |  |  |  |  |  |
| Negative | 26 (32.9%) | 53 (67.1%) | 1.036 | 0.309 |  |
| Positive | 1 (14.3%) | 6 (85.7%) |  |  |  |
| Data was shown as Mean ± SD. chi-squared test. **P* < 0.05. GC: gastric cancer. | | | | |  |

| **Supplementary Table S2** Primers for qRT-PCR analysis. | | |
| --- | --- | --- |
| Gene name | Forward primer | Reverse primer |
| Homo GAPDH | 5'-AGAAGGCTGGGGCTCATTTG-3' | 5'-AGGGGCCATCCACAGTCTTC-3' |
| Homo KIAA1429 | 5'-ATGCTGATGGGGAGAAAGAAG-3' | 5'-CAGGAGAAATGGGCTCAAAGTA-3' |
| Homo MMP-2 | 5'-TTGACGGTAAGGACGGACTC-3' | 5'-ACTTGCAGTACTCCCCATCG-3' |
| Homo MMP-9 | 5'-TGGCATCCGGCACCTCTATG-3' | 5'-GCATCG TCCACCGGACTCAA-3' |
| Homo RASD1 | 5'-CGACTCGGAGCTGAGTATCC-3' | 5'-GCGGATGGAGTAGAACTTGC-3' |
| qRT-PCR: quantitative real-time PCR. | | |

**References**

1. Ren M, Zhou X, Gu M, et al. Resveratrol synergizes with cisplatin in antineoplastic effects against AGS gastric cancer cells by inducing endoplasmic reticulum stress‑mediated apoptosis and G2/M phase arrest. *Oncol Rep.* 2020;44(4):1605-1615.
